# Can Immunogenic Chemotherapies Relieve Cancer Cell Resistance to Immune Checkpoint Inhibitors?

**DOI:** 10.3389/fimmu.2019.01181

**Published:** 2019-05-29

**Authors:** Thaiz Rivera Vargas, Lionel Apetoh

**Affiliations:** ^1^INSERM, U1231, Dijon, France; ^2^Faculté de Médecine, Université de Bourgogne Franche Comté, Dijon, France

**Keywords:** chemotherapy, checkpoint, immunomodulation, cancer, T cells

## Abstract

The unprecedented clinical activity of checkpoint blockade in several types of cancers has formally demonstrated that anti-tumor immune responses are crucial in cancer therapy. Durable responses seen in patients treated with immune checkpoint inhibitors (ICI) show that they can trigger the establishment of long-lasting immunologic memory. This beneficial outcome is however achieved for a limited number of patients. In addition, late relapses are emerging suggesting the development of acquired resistances that compromise the anticancer efficacy of ICI. How can this be prevented through combination therapies? We here review the functions of immune checkpoints, the successes of ICI in treating cancer and their therapeutic limits. We discuss how conventional cancer therapies can be properly selected to set up combinatorial approaches with ICI leading to treatment improvement. We finally summarize clinical data showing the ongoing progress in cancer treatment involving ICI and chemotherapy combination strategies.

## Introduction

Immune cells prevent the outgrowth of cancer cells before they form a clinically detectable tumor (1). Thus, while chronic inflammation supports cancer cell growth, defective immune responses also contribute to tumor formation and development. The complex relationships between immune cells and tumor cells are underscored by the paradoxical role of the immune system during the course of tumor progression. During the early stages of tumor development, immune cells eliminate cancer cells. However, resistant clones eventually develop in the tumor microenvironment and tumors can not only become resistant to the immune driven cytotoxicity but immune responses are also subverted to further promote tumor growth (2). This concept of “cancer immunoediting” pioneered by Robert Schrieber has been elegantly reviewed (2).

Downregulation of effector T cell function is one of the mechanisms used by cancer cells to escape recognition and elimination by the immune system. This results in the tumor microenvironment in the emergence of dysfunctional T cells, with a progressively compromised ability to secrete effector cytokines and kill cancer cells (3–8). Dysfunctional T cells can stem from functional effector cells, which gradually lost their effector function due to chronic activation through the T cell receptor (TCR) (3, 9). This is for instance illustrated in mice with persistent chronic viral infections, where virus-specific CD8 T cells undergo dysfunction, which can however be reversible during the early stages of infection (10, 11). The induction of T cell dysfunction is also a physiological process that prevents the development of autoimmunity. This was exemplified in mouse studies where interfering with T cell dysfunction led to severe CD8 T cell-driven immunopathologies (12, 13). Thus, tumors subvert physiological immune responses that normally prevent excessive immune activation to further promote their growth.

Dysfunctional T cells can be characterized by the concomitant expression of several inhibitory receptors (IR), a reduced secretion of effector cytokines, such as IL-2, TNFα, and IFNγ as well as altered cell metabolism and a markedly different transcriptional profile (5, 6, 8, 14–18). Accordingly, compounds blocking IR engagement and signaling, i.e., immune checkpoint inhibitors (ICI), were developed and led to durable disease control in advanced cancers (19, 20). These successes ultimately translated into the clinic. Indeed, antibodies directed the immune checkpoints CTLA-4 (cytotoxic T lymphocyte-associated protein 4) and PD-1 (programmed cell death protein-1) were shown to prolong the survival of cancer patients suffering from metastatic cancers. While these achievements were initially noted in patients with metastatic melanoma (21, 22), they therapies are now approved to treat multiple types of cancers (23–26).

Despite the tremendous success of ICI, some tumors are resistant to these therapies while others gain resistance during the course of the treatments, thereby compromising their efficacy (27). Generally, immunogenic tumors with limited size and strong T cell-infiltration respond to checkpoint blockade. Therefore, increasing tumor immunogenicity and T cell presence in the tumor bed while reducing tumor burden are key factors to improve disease outcome. Because it has been proposed that some chemotherapies could trigger immune activation (28, 29), these drugs may act valuable partners with checkpoint blockade and combinatorial approaches are warranted. We first discuss the consequences of the use of ICI on immune and cancer cells. We then review key chemotherapy properties that are able to improve patient's response to checkpoint blockade and discuss how this knowledge could be exploited to translate immunotherapy-based approaches into more successful therapeutic combinations.

## The Success Story of Checkpoint Blockade-Based Cancer Therapies

### Cancer Treatment: A Tightrope Walker on the Fine Line Between T Cell Activation and T Cell Dysfunction

A deep knowledge of the mechanisms that drive T cell activation is required to design improved therapeutics unleashing T cell anticancer properties without triggering T cell dysfunction. Given the recent clinical success of ICI therapies, this area of research has attracted not only academic scientists but also pharmaceutical industries. T cell activation relies on TCR-driven signals, cytokines as well as co-stimulatory signals that will ultimately drive the outcome following T cell stimulation (30–32). Co-signaling receptors present on T cells can either convey stimulatory or inhibitory signals. The expression of these co-signaling receptors is dependent on the environment surrounding the T cells. Thus, neighboring cells harboring ligands of these receptors will profoundly affect T cell activation. This is thoroughly documented for antigen presenting cells (APCs), which profoundly shape T cell activation (33–35). The outcome of T cell activation thus results from the integration of activating and inhibitory signals.

From a therapeutic perspective, strategies altering T cell costimulation have been successfully exploited to restore T cell activation and anticancer immune responses. The initial characterization of CTLA-4 as molecule negatively regulating T cell activation has indeed prompted the development of antibody-mediated therapies that disrupt T cell dysfunction, reinvigorate effector T cells and dampen the activity of regulatory T cells, resulting in anticancer immunity (22, 36, 37). Manipulation of the PD-1 signaling pathway has similarly led to remarkable activation of anticancer T cells [reviewed in (38)]. These strategies have been successfully implemented into the clinic and the administration of antibodies preventing the engagement of PD-1 in cancer patients has resulted in response rates ranging from 20 to 90% in different cancer types (21, 23–26, 38–42). These remarkable results led to the routine use of these therapies to treat patients suffering from different cancer types, including metastatic melanoma and non-small-cell lung cancer (NSCLC). ICI treatments thus represent a major advance in cancer immunotherapy, which was further underscored by the scientific community that awarded of the Nobel Prize in Physiology or Medicine to the two researchers who identified CTLA-4 and PD-1 in 2018. However, as discussed below, other cancers, such as microsatellite stable (MSS) colon cancers respond poorly to ICI therapies, highlighting the need to pursue efforts to decipher the mechanisms explaining the resistance of some cancers to ICI therapies.

### Mechanisms of Resistance to Immune Checkpoint Inhibitors

Patients with melanoma and Hodgkin lymphoma exhibit the best response rates following treatment with ICI therapies (38). About 20% of melanoma patients who received anti-CTLA-4 (ipilimumab) still exhibit a complete response 10 years after treatment initiation (36). Likewise, melanoma patients administered anti-PD-1 (pembrolizumab) featured a 3-year response rate of 33% (39). The added value of combination therapies of ICI has been tested in metastatic melanoma patients. Upon combined treatment with anti-CTLA-4 and anti-PD-1, a high response rate of 58% was achieved but was associated with severe toxicity (40, 41). Patient treatment with ICI therapies can lead to various outcomes. Unfortunately, even in cancers susceptible to ICI therapies, a substantial fraction of patients will not respond to treatment. Conversely, other patients exhibit a desirable response that will be long-lasting and lead to a complete response. However, some patients, who initially benefit from the therapy, eventually become resistant to treatment, resulting in cancer outgrowth (27, 43–45).

The observation that about two-third of patients do not respond to the administration of ICI given as a monotherapy prompted scientists and physicians to identify factors able to predict treatment efficacy (26, 40, 46–50). It now established that one of the important factors in determining the clinical response to ICI is the number of tumor mutations (51–57). This is illustrated for instance by the better response to anti-PD-1 therapy of colorectal cancers with microsatellite instability compared to MSS colorectal cancers. An additional parameter that affects ICI clinical efficacy is the presence and level of expression of the ligand of the targeted checkpoint by tumor cells and immune cells infiltrating the tumor bed (26, 58, 59). Finally, patients featuring an increased frequency of proliferating CD8 T cells following treatment with ICI was associated with a beneficial response (60). Thus, the defective generation of effector and memory CD8 T cell responses following ICI treatments can compromise their efficacy (27, 45, 61, 62). The quality of T cell responses will critically depend on the availability of tumor-derived neoantigens that will trigger the development of tumor-specific T cells (45). In addition, overcoming the immunosuppression present in the tumor microenvironment is essential for effective and long-lasting T cell responses to be maintained following treatment (43, 63, 64). Combination therapies should thus be designed with the aim to reinforce tumor immunogenicity, alleviate immunosuppression, and enhance T cell trafficking and memory.

## Chemotherapy to Improve Checkpoint Blockade-Based Cancer Therapies

**Figure 1:**
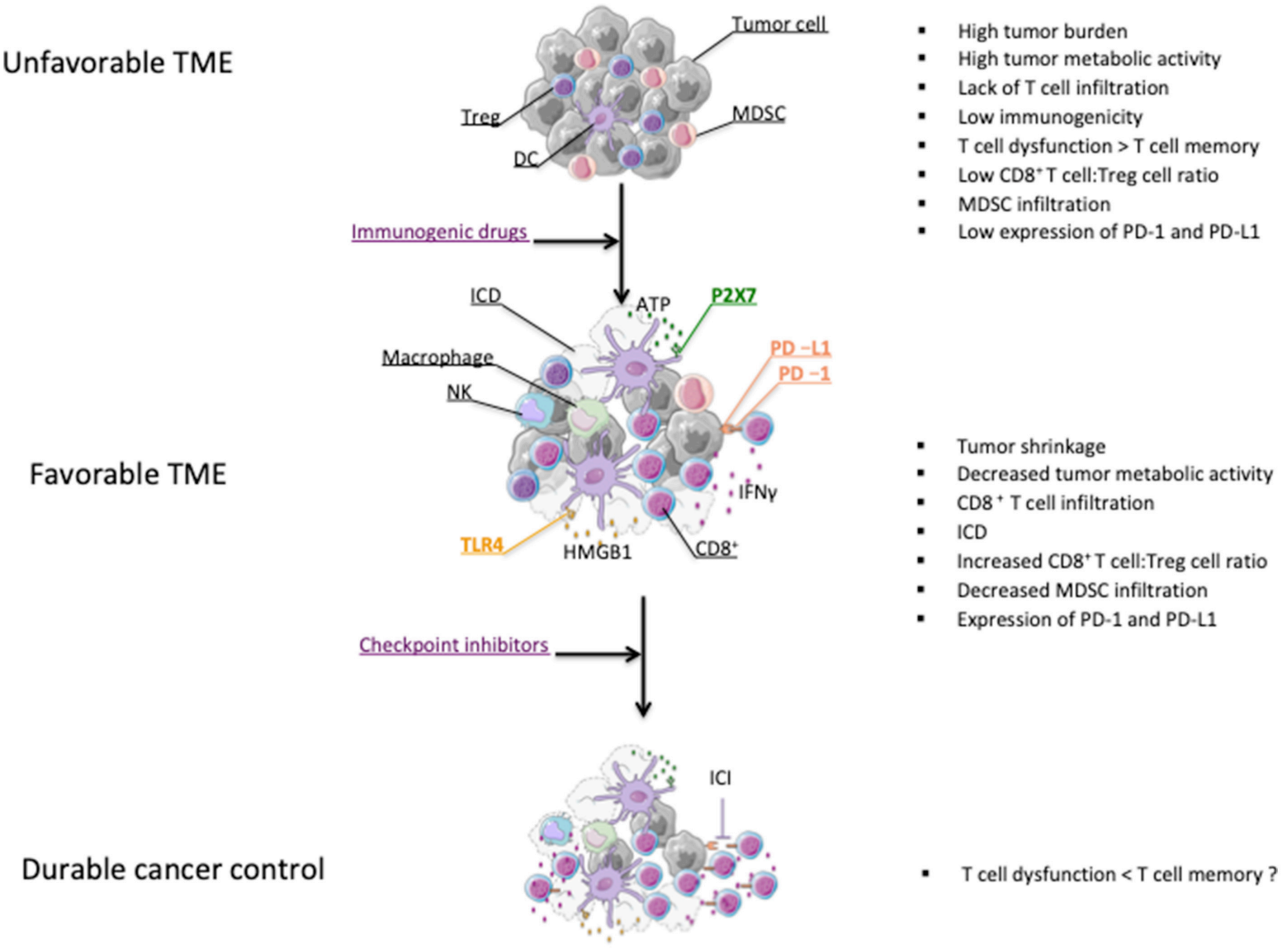
Effect of chemotherapy on the modification of the tumor microenvironment (TME). Chemotherapies leading to tumor shrinkage can shift the tumor microenvironment from tumor promoting to tumor suppressive (38). Chemotherapeutic drugs can induce ICD leading to the release of danger signal molecules that will contribute to the induction of anticancer immunity (74). Chemotherapy can also enhance the CD8^+^ T cell:Treg cell ratio and eliminate myeloid-derivative suppressive cells (MDSCs) (75–77). Some chemotherapy treatments promote the activation of CD8 T cells as well as PD-L1 expression on tumor cells (75). All these effects may partly explain why chemotherapies and ICI therapies can possibly synergize for durable cancer control (75, 78).

Most chemotherapeutic agents are involved in disrupting DNA replication leading to apoptosis of dividing cells (65). Mechanisms of direct tumor killing by chemotherapy involve DNA damage, inhibition of DNA replication, and prevention of mitosis (66). The cytotoxic properties of chemotherapy against dividing cells, which include immune cells, explain their well-known ability to trigger immunosuppression. This knowledge has led to implement treatments like high-dose cyclophosphamide to alleviate the course of severe autoimmune diseases (67). However, preclinical findings reported in the last fifty years suggested that chemotherapies could favor anti-tumor immunity [(68) and reviewed in (28, 29, 69)]. These beneficial effects on anticancer immunity were not only attributable to chemotherapy directly affecting tumor cells, thereby enhancing tumor cell immunogenicity, but also through the direct killing of immunosuppressive cells (70–73). All these properties provide a solid rationale for strategies combining checkpoint inhibitors and chemotherapy agents (Figure 1).

### Chemotherapy Enhances the Development of T Cell-Dependent Anticancer Responses

Some chemotherapeutics like doxorubicin, cyclophosphamide and oxaliplatin can induce an immunogenic form of tumor cell death that will contribute to the development of T cell-dependent anticancer responses. Immunogenic cell death (ICD) is characterized by a series of molecular events responsible for the induction of anticancer immunity. The first feature of ICD is the cell surface expression of calreticulin, which is responsible for the phagocytosis of tumor cells undergoing ICD by dendritic cells (DC) (79). Another key event dictating the immunostimulatory activity of ICD is the release of the High mobility group box 1 (HMGB1) protein that is essential for an efficient cross-presentation of tumor-derived antigens by DC (80). In addition, HMGB1, together with ATP, which is liberated following tumor cell insult, will drive the secretion of IL-1β from DC, thereby enhancing CD8 T cell activation (81). We have investigated the importance of this immunoadjuvant pathway in humans by studying a retrospective cohort of breast cancer patients with lymph node involvement and treated with anthracyclines and radiotherapy. We found that patients with polymorphisms affecting the signaling of TLR4 and the high affinity ATP receptor, P2X7, were associated with a faster time to progression, thereby suggesting that anticancer immune responses driven through ICD are relevant in humans (80–82) (Figure 1).

In the context of an unfavorable TME two major questions are: 1) How to restore T cell infiltration and anticancer immunity? Is this process able to sensitize tumors to ICI therapy? To address this, Pfirschke et al. used a conditional lung adenocarcinoma mouse model with Kras and Trp53 mutations (KP model) (78). This model features molecular characteristics that mimic the development of human lung cancer. An important feature in this model is that T cells weakly infiltrate the TME. Accordingly, in this context anti-PD-1 treatment given alone failed to delay tumor progression. The authors then introduced neoantigens to sensitize the tumors to ICI therapy. For this, they generated KP-OVA mice, whose tumors expressed ovalbumin-derived peptides. However, even in the presence of these exogenous antigens, tumors from KP-OVA mice were resistant to combined treatment with anti-PD-1 and anti-CTLA-4 (78). The authors then screened different chemotherapies to test their anticancer efficacy in this model. Among several NSCLC chemotherapeutics (paclitaxel, docetaxel, carboplatin, mitoxantrone), they found that the combination of oxaliplatin and cyclophosphamide significantly increased nuclear HMGB1 staining in tumor nodules. While the combination paclitaxel-carboplatin failed to suppress cancer progression, oxaliplatin-cyclophosphamide (OX-CTX) combination controlled tumor growth. Accordingly, anticancer immune responses were enhanced in the lungs of the treated mice, as illustrated by increased presence of CD8 T cells along with reduced Treg frequency. Importantly, proliferating effector T cells were noted in the TME following the combined treatment (78). This chemotherapy-driven activation of adaptive immune responses was critical for the beneficial effect of the treatment as illustrated by the inability of Rag2^−/−^ KP mice, which lack T and B cells, to control cancer growth upon OX-CTX treatment. The authors also tested the involvement of TLR4 in the ability of KP mice to respond to combined chemotherapeutic treatment. They found that mice deficient for TLR4 featured a decreased ability to resist KP tumor progression. Thus, TLR4 is required for the effective induction of T-cell dependent anticancer immunity in this genetic lung cancer tumor model following treatment with chemotherapy. These results are in line with our pioneering studies obtained using transplantable tumor models indicating that TLR4 dictates the immunogenicity of chemotherapy (80).

In addition to noting tumor-specific CD8 T cell infiltration in the lungs of KP-OVA mice upon combined treatment with OX-CTX, the authors found that following this treatment these tumor-specific cells expressed PD-1. Moreover, cells of the neighboring TME expressed PD-L1. This suggested that in that context the efficacy of the treatment could possibly be dampened by the engagement of the PD-1/PD-L1 pathway in tumor-specific CD8 T cells. To test this, KP-OVA mice were treated with anti-CTLA-4 and anti-PD-1, OX-CTX or the combined chemo-immunotherapeutic treatment. They found that the latter treatment featured a superior ability to control tumor growth compared to the monotherapies. Collectively, these results suggest that chemotherapy can restore T cell infiltration and activation even in the non-immunogenic KP lung cancer model and sensitize tumors to subsequent and/or concomitant treatment with ICI (78) (Figure 1).

In line with Pfirschke's work, we recently showed that 5-Fluorouracil plus Oxaliplatin (Folfox), in contrast to monotherapies, drove complete tumor cures in two mouse colorectal cancer models when combined to anti-PD-1 treatment, thereby suggesting that Folfox administration renders colorectal tumors sensitive to PD-1 blockade (75). Interestingly, the ability of Folfox to synergize with anti-PD-1 therapy was directly associated with the Folfox-driven induction of IFNγ-producing CD8 T cells as well as of enhanced PD-L1 expression on tumor cells *in vivo*. In line with this, we found in the CT26 colon adenocarcinoma model that Mitomycin C, which does not promote tumor PD-L1 expression on tumor cells *in vivo*, failed to synergize with anti-PD-1 treatment. Thus, the therapeutic success of the Folfox/Anti-PD-1 combination therapy is linked to the Folfox-driven induction of an anticancer immune response that drives tumor adaptive immune resistance through the PD-1/PD-L1 pathway (75).

We have further studied the molecular events leading to the development of anticancer immunity following Folfox administration. We first identified that Folfox drove the induction of TLR4-dependent immunity, resulting in CD8 T cell activation. In addition, this combined treatment not only induced the depletion of Myeloid derived suppressor cells (MDSCs) *in vivo* but also induced CD8 T cells expressing higher levels of the transcription factor T-bet compared to mice treated with monotherapies. We have investigated the relevance of this observation using mice lacking conditionally the expression of T-bet in CD4 and CD8 T cells. We noted that in these mice the therapeutic effect of Folfox against MC38 colon carcinomas was lost, indicating that T-bet expression in this context was required for the induction of T cell-dependent anticancer immune responses. We also unraveled the signaling pathway driving PD-L1 expression on tumor cells *in vivo* following Folfox administration. Using either T cell-deficient nude mice, mice depleted of CD8 T cells as well as mice receiving IFNγ neutralizing antibodies, we identified IFNγ-secreting CD8 T cells as a major driver of PD-L1 tumor expression *in vivo* following Folfox treatment. While we were unable to rule out a contribution of other IFNγ-producing cells in our observations, it is notable that we identified a strong correlation between the ability of different chemotherapies to induce CD8 T cell infiltration in the tumor *in vivo* and the induction of PD-L1 tumor expression. Overall, Folfox triggers a CD8 T cell-dependent anticancer immune response that in turn drives tumor PD-L1 expression, which thus acts as an adaptive resistance mechanism to the combined therapy. This resistance is successfully overcome by the addition of ICI therapy and our results therefore prompt for the combination of immunogenic drugs with ICI (75) (Figure 1).

Successful chemo-immunotherapy combinations involving the use of ICI are not restricted to antibodies targeting CTLA-4 or PD-1. Indeed, De Mingo Pulido et al. have just reported in mouse models of breast cancer that anti-Tim-3 treatment could improve the anticancer effect of paclitaxel (PTX) while anti-PD-1 therapy could not do so (83). Tim-3 was initially characterized as an immunoglobulin expressed on highly polarized Th1 cells (84). We and others subsequently showed that Tim-3 was also present on dysfunctional CD8 T cells in mouse and human tumors (17, 18). These findings were relevant as blockade of Tim-3 and PD-L1 *in vivo* could prevent tumor outgrowth (17). Interestingly, while Tim-3 was weakly expressed on CD8 T cells from mouse MMTV-PyMT tumors, the combined therapy induced CD8 T cell anticancer immunity (83). In fact, myeloid cells from both mouse and human tumors expressed Tim-3 and combined therapy with PTX and anti-Tim-3 triggered CXCL9 expression on DCs, possibly enhancing DC/T cell interactions and resulting in anticancer immunity. Accordingly, in human breast cancer patients, CXCL9 expression correlates with response to neoadjuvant chemotherapy (83). Thus, Tim-3 represents a molecular target, which can be exploited in the setting of combinatorial treatments relying on chemotherapy.

During ICD certain chemotherapies can also induce the release of various danger signals. For instance, DNA leakage into the cytosol can lead to the engagement of cytosolic DNA sensors, which will trigger the secretion of type I interferon from tumor cells, thereby leading to the induction of anticancer immune responses (74, 85). Chemotherapy also favors the generation of mutations in cancer cells, thereby increasing their antigenicity and rendering them more sensitive to ICI therapy (54, 86). Some chemotherapies will enhance tumor expression of MHC molecules, which enhances their ability to present tumor antigens and thus immunogenicity (85, 87, 88). Drugs like CTX can also drive lymphopenia, which can be exploited therapeutically in the context of combination therapies to drive immune activation and anticancer immunity (89–92). Thus, chemotherapy can be an attractive partner of ICI that can overcome ICI resistance due to insufficient anti-tumor T cell generation.

### Chemotherapy Resets the TME to Favor T-cell Effector Function

Immunosuppressive cells present in the TME compromise the anticancer efficacy of ICI. Mouse studies have documented that myeloid cells, including tumor-associated macrophages (TAMs) and MDSCs, as well as Tregs and Th2 lymphocytes can contribute to the repression of anticancer T cell responses following ICI administration (27, 43, 45). Accordingly, preventing the accumulation of these immunosuppressive cells in the TME enhances the efficacy of ICI therapy (93, 94). The mechanisms accounting for these observations are progressively being unraveled. It was for instance shown that TAMs featured the ability to capture anti-PD-1 antibodies, which are thus no longer able to target CD8 T cells (95). MDSC are likewise able to suppress immune responses because of their immunosuppressive enzymes like indolamine-2,3-dioxygenase and arginase 1 that will dampen DC and T cell effector functions (96, 97).

Chemotherapy has the ability to eliminate immunosuppressive cells from the TME. CTX was shown to have a remarkable ability to eliminate Treg cells (98). These results have been extended to the human setting. Treg frequency in cancer patients treated with repeated, lose-dose cyclophosphamide was indeed reduced, underscoring the relevance of these observations initially made in preclinical studies (99, 100). We and others have also demonstrated that MDSC could be selectively targeted by some chemotherapies (101). Initially, gemcitabine was identified in mouse studies as a drug capable of eliminating MDSC in tumor-bearing mice *in vivo* (102). MDSC elimination using gemcitabine improved CD8 T cell functions and favored tumor control (102). Upon screening different chemotherapies to test their ability to eliminate MDSC *in vivo*, we not only could confirm the results of Li et al. but also noted that 5-Fluorouracil (5-FU) efficiently killed MDSC (76). In CT26 tumor-bearing mice, 5-FU was indeed able to eliminate MDSC without strongly affecting the frequency of T, B, or NK cells (76). We found that the high sensitivity of MDSC to 5-FU could be attributed to their low expression of Thymidylate synthase. In the setting of breast cancer, doxorubicin was likewise shown to target MDSC, leading to an improved efficacy of T cell adoptive transfer (103). Overall, chemotherapy has the ability to profoundly affect the TME and drive immune activation through the direct elimination of immunosuppressive cells, thereby enhancing the activity of ICI therapies.

### Does Chemotherapy Influence the Formation of T-cell Memory?

Most if not all tumor-infiltrating lymphocytes feature memory T cell phenotypes (14, 104–106). CD8 T cells infiltrating the TME lack the expression of naïve T cell markers and instead express surface marker proteins found on activated and dysfunctional T cells (14). Thus, TILs harvested from cancer patients, which were shown to induce potent anticancer responses after *in vitro* expansion and reinfusion (107), are actually stemming from memory T cells (108–111). These observations are significant as an explanation for the ability of ICI therapies to induce anticancer responses is that these treatments actually reactivate preexisting immune responses. In addition, the most striking successes of ICI therapies lie in their abilities to induce long-lasting anticancer immune responses, resulting in long-term complete responses. This evidence altogether suggests that ICI therapies could affect the generation of anticancer memory T cells. This would actually be in line with previous preclinical and clinical observations showing CD8 T cell expansion following interference with the PD-1/PD-L1 pathway (58). Finally, this would also be congruent with recent preclinical findings obtained on isolated CD8^+^ TILs following combined treatment with ICI (112). Indeed, combined therapies induced profound transcriptional changes in PD-1 negative TILs that exhibited a memory precursor-like phenotype as well as effector functions in various cancer models (112). However, the induction or restoration of memory T cell responses in patients with high tumor loads is challenging (60), underscoring the need to design alternative strategies to improve therapeutic outcome. In this regard, the knowledge that chemotherapy-induced ICD shapes the quality of T cell responses (113, 114), will be key to combine selected chemotherapeutic agents with immunomodulation to generate potent memory T cell responses.

## Clinical Activity of Therapeutic Strategies Combining ICI and Chemotherapy

The discoveries relating to molecular rationales for combinatorial approaches involving ICI and chemotherapy led to several clinical studies. We here discuss a few examples underscoring the relevance of combining ICI with chemotherapy. In NSCLC, the efficacy of the combination of anti-PD-1 (pembrolizumab) with chemotherapy was evaluated in the KEYNOTE-021 phase II clinical trial. Results revealed that the combination of anti-PD-1 treatment with chemotherapy (carboplatin and pemetrexed) led to significantly higher response rate in comparison to chemotherapy alone (Table 1) (116). The addition of immunomodulation together with chemotherapy was not associated with enhanced toxicity (116, 127). An updated analysis confirmed a beneficial effect both in terms of improved response rate and progression free survival (127). This led the FDA to grant accelerated approval for this combination therapy to treat metastatic NSCLC patients who have not been previously received chemotherapy and without targetable mutations. Interestingly, higher tumor PD-L1 expression (≥50%) was associated with an enhanced response rate of 80% but given the relatively low numbers of patients studied and observations that patients with <1% PD-L1 expression and with ≥1% PD-L1 expression featured comparable response rate of 54 and 57%, respectively, this study did not conclude on any link between combined treatment efficacy and tumor PD-L1 expression (116). Nevertheless, these observations were of clinical relevance because they provided a therapeutic option to NSCLC patients harboring below 50% tumor PD-L1 expression. Indeed, the efficacy of anti-PD-1 (pembrolizumab) treatment given alone was initially confirmed in patients featuring above 50% PD-L1 expression (24, 128). The KEYNOTE-189 phase III trial subsequently tested the benefit of the addition of anti-PD-1 therapy to chemotherapy in nonsquamous NSCLC (118). Results revealed, independently of the tumor PD-L1 status, that the addition of anti-PD-1 antibody to chemotherapy led to improved overall survival and progression free survival compared to chemotherapy alone. Full approval was then granted by the FDA to anti-PD-1 treatment (pembrolizumab) in combination with chemotherapy for the treatment of NSCLC patients. Whether this combination can also benefit patients with squamous NSCLC is currently being tested in a phase 3 trial (KEYNOTE-407). Overall, the addition of ICI to chemotherapy has changed the standard of care of metastatic lung cancer patients.

**Table 1:** Examples of completed and ongoing clinical trials evaluating immunomodulation using anti-PD-1, anti-PD-L1, or/and anti-CTLA-4 in combination with chemotherapy.

**Study**	**Tumor type**	**Endpoints**	**References**
**ANTI-PD-1**			
**KEYNOTE 021 (phase 1)**			
Cohort A: Pembro + CBDCA + PTX → Pembro	NSCLC	Cohort A: ORR: 48%Median PFS: 10.3 months	(115)
Cohort B: Pembro + CBDCA + PTX + BEV → Pembro + BEV		Cohort B: ORR: 56%Median PFS: 7.1 months	
Cohort C: Pembro + CBDCA +PEM → Pembro + PEM		Cohort C: ORR: 75%Median PFS: 10.2 months	
**KEYNOTE 021 (phase 2)**			
Pembro + CBDCA + PEM → Pembro + PEM	NSCLC	ORR: 56.7%PFS: 24.0 months	(116)
CBDCA/PEM → PEM		ORR: 30.2%PFS: 9.3 months	(117)
**KEYNOTE 189 (phase 3)**			
Pembro + PLAT + PEM → Pembro + PEM	NSCLC	OS (12 months): 69.2%PFS: 8.8 months	(118)
Placebo + PLAT + PEM → PEM		OS (12 months): 49.4%PFS: 4.9 months	
**PEMBRO-PLUS (phase 1b)**			
Pembro with either GEM, GEM + DOCE, GEM + Nab-PTX, GEM + VINO, IRINO or DOXO	Solid tumors	8 partial responses	(119)
**CHECKMATE 012 (phase 1)**			
Nivo +GEM + CIS → Nivo	NSCLC	PFS: 5.7 months OS: 11.6 months	(120)
Nivo + PEM + CIS → Nivo		PFS: 6.8 months OS: 19.2 months	
Nivo (10 mg/kg) + CBDCA + PTX → Nivo		PFS: 4.8 months OS: 14.9 months	
Nivo (5 mg/kg) + CBDCA + PTX → Nivo		PFS: 7.1 months	
**ANTI-PD-L1**			
**NCT01633970 (phase 1)**			
Atezo + CBDCA + PTX → Atezo	NSCLC	ORR: 36%, PFS: 7.1 months, OSS: 12.9 months	(121)
Atezo + CBDCA/PEM → Atezo + PEM		ORR: 68%, PFS: 8.4 months, OS: 18.9 months	
Atezo + CBDCA + Nab-PTX → Atezo		ORR: 46%, PFS: 5.7 months, OS: 17.0 months	
**NCT02367781 IMpower 130 (phase 3) (NSCLC nonsquamous)**Atezo + CBDCA + Nab-PTX → Atezo	NSCLC	Primary: OS and PFS	
**NCT02367794 IMpower 131 (phase 3) (NSCLC Squamous)**Atezo + CBDCA + PTX → Atezo		Secondary: ORR	
**NCT02657434 IMpower 132 (phase 3) (NSCLC nonsquamous)**Atezo + PEM/ CBDCA (or CIS) → Atezo + PEM			
**NCT02366143 IMpower 150 (phase 3)**			
CBDCA + PTX + BEV	NSCLC	PFS: 6.8 months	(122)
Atezo + CBDCA + PTX + BEV		PFS: 8.3 months	
**NCT03164616 POSEIDON (phase 3)**			
Durval + Tremeli + chemotherapy	NSCLC	Primary: OS and PFS	
Durval + chemotherapy		Secondary: ORR	
Chemotherapy			
**NCT02735239 (phase 1/2)**			
Durval with chemotherapy	Esophageal cancer	Primary: safety	
		Secondary: ORR, PFS, OS	
**NCT03317496 (phase 2)**			
Ave + CBDCA/PEM	Solid tumors	Primary: OR	
Ave+ GEM/CIS		Secondary: PFS, OS	
**ANTI-CTLA-4**			
**Phase 3**			
Ipili+ CBDCA + PTX	NSCLC	OS: 13.5 months PFS: 5.6 months	(123)
Placebo + CBDCA + PTX		OS: 12.4 months PFS: 5.6 months	
**Phase 2**			
Ipili + temozolomide	Melanoma	6-month PFS: 45% median OS: 24.5 months	(124)
**Phase 3**			
Ipili + ETOP and PLAT	SCLC	OS: 11 months PFS: 4.6 months	(125)
Placebo + ETOP and PLAT		OS: 10.9 months PFS: 4.4 months	
**Phase 2**			
Ipili + DACARB	Melanoma	High toxicities noted	(126)
**NCT03202758 (phase 1b/2)**			
Durval + Tremeli + FOLFOX	Colon cancer	Phase 1b: Safety	
		Phase 2:	
		Primary: PFS	
		Secondary: OS	

*Atezo, Atezolizumab; Ave, Avelumab; Durval, Durvalumab; Ipili, Ipilimumab; Nivo, Nivolumab; Pembro, Pembrolizumab; Tremeli, Tremelimumab; BEV, Bevacizumab; CBDCA, Carboplatin; CIS, Cisplatin; DACARB, Dacarbazine; DOC, Docetaxel; DOXO, Doxorubicin; ETOP, Etoposide; FOLFOX, 5-Fluorouracil and Oxaliplatin; GEM, Gemcitabine; IRINO, Irinotecan; Nab-PTX, Nab-Paclitaxel; PEM, Pemetrexed; PLAT, Platinum; PTX, Paclitaxel; VINO, Vinorelbine; ORR, Overall Response Rate; OS, Overall survival; PFS, Progression-free survival*.

Therapeutic successes have been achieved when combining other ICI with chemotherapy. In the phase 1 CHECKMATE-012 trial, NSCLC patients received first-line therapy with a combination of anti-PD-1 (Nivolumab) and chemotherapy associations (120). While enhanced toxicity was observed, a beneficial activity of the combination was noted especially for the paclitaxel-carboplatin association. Other combinations of ICI with chemotherapy agents are being evaluated in clinical trials in other cancer types (Table 1). Whether pembrolizumab efficacy in combination with chemotherapy can be extended to other cancer types in addition to lung cancer will be investigated in a phase I/II study (NCT02331251) that will include different chemotherapies and solid tumors, including sarcoma, breast and ovarian cancer. Likewise, the ability of Pembrolizumab to bring additional benefit to chemotherapy will also be searched in the KEYNOTE-062 study in gastric cancer patients.

Importantly, interfering with the PD-L1/PD-1 pathway in combination with chemotherapies using PD-L1 inhibitors has recently led to important clinical achievements. The Impower 150 study underscored the superior efficacy of the addition of atezolizumab to bevacizumab plus chemotherapy in metastatic nonsquamous NSCLC (122). These results were independent of the PD-L1 status of the tumor. The success of this study has led to the approval of this combined therapy in late 2018 for the treatment of metastatic non-squamous NSCLC with atezolizumab with chemotherapy and bevacizumab as first-line treatment. Other phase III studies currently assess the benefit of atezolizumab with different chemotherapy combinations in lung cancer (Table 1). Likewise, the therapeutic benefit of combining other anti-PD-L1 antibodies to chemotherapy in various malignancies is currently being evaluated [Table 1 and reviewed in (129)]. For instance, based on our initial preclinical findings suggesting the ability of chemotherapy to synergize with the blockade of the PD-1/PD-L1 pathway in colon cancer [(75) and discussed above], the MEDITREME trial was initiated at the anticancer center Georges François in Dijon, France. The aim will be to determine the safety and efficacy of the anti-PD-L1/anti-CTLA-4 treatment given in combination with chemotherapy (FOLFOX) (Table 1).

While these different examples illustrate the successes and the possibilities of combining chemotherapy with ICI, even leading in some situations in changing the routine clinical practice in non-squamous NSCLC, it should be noted that some combination therapies have yielded disappointing results. It is indeed notable that in lung cancer the targeting of CTLA-4 combined to chemotherapy has brought no additional therapeutic benefit over chemotherapy treatment given alone in phase 3 clinical trials (Table 1). Thus, while some exciting novel treatment options have been provided by combination therapies using ICI and chemotherapy, the identification of additional successful combinations is warranted as discussed below.

## Conclusion

Some chemotherapies have been coined with the term “immunogenic,” i.e., the ability to induce an immune response. The latter can stem from the elimination of suppressive cells, the induction of ICD and/or of effector T cell functions, and possibly lead to tumor eradication (Figure 1). It should be re-emphasized that the ability of chemotherapies to induce ICD is only one of the mechanisms by which they can promote immunity. For instance the drug cisplatin, despite its weak ability to induce ICD, was shown to be immunogenic. Indeed, cisplatin enhances antitumor adaptive immunity by increasing tumor cell killing by CD8 T cells (130, 131). Because high dose chemotherapy can promote immunosuppression as discussed above, a careful selection of the optimal chemotherapy dose to be administered is required to harness the immunogenic properties of chemotherapies while minimizing their immunosuppressive effects (130). Can immunogenic chemotherapies then be used to relieve cancer cell resistance to immune checkpoint inhibitors? While the answer in preclinical models clearly seems to be yes [(75, 78) and reviewed in (129)], the translation of these findings to the clinic has been challenging. While combined treatments with chemotherapy and ICI targeting PD-1/PD-L1 signaling have been effective in human non-squamous NSCLC, disappointing results were obtained when administering chemotherapy and anti-CTLA-4 (Table 1). The lack of relevant biomarkers indicative of anti-CTLA-4 treatment efficacy may explain the difficulties to harness the full potential of this strategy (132). Despite these hurdles, the recent proof-of-principle study showing the ability to successfully combine other ICI such as Tim-3 with chemotherapy opens up a myriad of novel possibilities to be subsequently tested in a clinical setting (83). Identifying and validating additional biomarkers indicative of the efficacy of ICI will help to optimize the most effective ICI—chemotherapy combinations to be given to cancer patients.

## Author Contributions

TRV drafted the review and prepared the figure. LA revised and edited the review.

### Conflict of Interest Statement

LA has performed consultancy work for Roche, Merck, and Bristol-Myers Squibb. LA is a recipient of a research grant from Sanofi. The remaining author declares that the research was conducted in the absence of any commercial or financial relationships that could be construed as a potential conflict of interest.
